# What Lies Below: A Theory of Planned Behavior Study of Septic System Owners’ Practices in the Attoyac Bayou Watershed

**DOI:** 10.1007/s00267-025-02367-z

**Published:** 2026-01-30

**Authors:** Emmanuel. C. Okolo, Audrey McCrary, Karissa Palmer, T. Allen Berthold, Holli R. Leggette

**Affiliations:** 1 Department of Agricultural Leadership, Education, and Communications, Texas A&M University, College Station, TX USA; 2 Texas Water Resources Institute, Texas A&M AgriLife, College Station, TX USA

**Keywords:** Theory of planned behavior, Watershed, *E. coli* in rural water systems, Septic system maintenance, Environmental public health

## Abstract

As populations increase, water quality is increasingly affected by failing septic systems that introduce harmful fecal bacteria (e.g., *E. coli*) into watersheds. Septic system owners play a vital role in reducing the impact of such bacteria. Therefore, our study aimed to examine factors that influence septic system owners’ decisions to improve septic system maintenance and protect watershed health in the Attoyac Bayou, located in East Texas. Using the theory of planned behavior, we addressed three research questions: (1) What are the characteristics of septic systems within the Attoyac Bayou watershed?; (2) How have septic system owners in the Attoyac Bayou watershed maintained their systems?; and (3) How do attitudes, subjective norms, and perceived behavioral control predict septic system maintenance behaviors? We sent a questionnaire to septic system owners in the Attoyac Bayou watershed that included questions about septic system characteristics, maintenance histories, and owners’ perceived norms, controls, attitudes, and intentions about septic systems. We found most septic systems in Attoyac Bayou are older conventional models with many owners lacking service contracts and reporting various times since the last pump out or inspection. While septic system owners generally demonstrate positive attitudes and confidence toward maintenance, our results revealed that attitude accounts for the greatest variance in maintenance behavior, with social norms and perceived behavioral control being less influential. Therefore, we recommend targeted messaging and interventions to reinforce positive attitudes, enhance perceived behavioral control, and promote consistent maintenance behaviors.

## Introduction

Access to safe water, sanitation, and hygiene is a fundamental need for human health. Yet, globally, an estimated 1.6 billion people will lack safe drinking water by 2030, and about 2.8 billion will lack safely managed sanitation due to declining investments in water quality and sanitation (United Nations, 2022). The impacts of poor water, sanitation, and hygiene contribute to the spread of disease, health risks from exposure to chemicals and contaminants, and higher mortality rates because of the effects of poor sanitation on mental well-being (World Health Organization, 2025). Therefore, because of rapid population growth, urbanization, and increased water needs in sectors like agriculture, industry, and energy, the demand for clean water continues to rise (Sustainable Development Goals, 2025).

Furthermore, the incessant degradation of coastal water quality, affecting urban, peri-urban, and rural watersheds, is a pressing concern (Khatri and Tyagi, 2014). This degradation, driven by natural processes and anthropogenic activities, has profound implications for the ecological network of social systems, the economy, and public health (Brewton et al., 2022). Key negative impacts of such degradation include increased harmful bacterial contaminations (HBC), eutrophication, and oxygen depletion in water bodies, largely due to excess nutrient effluents such as nitrogen and phosphorus (Sorenson et al., 2023). These impacts result in the loss of natural habitats for aquatic organisms and a significant decline in water quality, adversely impacting human health and socio-economic stability (Brewton et al., 2022). Septic systems have been shown to introduce some nitrogen and phosphorus into the water supply. (Sorenson et al., 2023). Additionally, scholars have also documented other factors contributing to watershed degradation, including weather conditions, rainfall in catchment areas, and specific watershed characteristics (Lapointe et al., 2012; Tran et al., 2015).

Scholars consider septic system failures as a threat to water quality degradation (Huffman et al., 2018; Wiegner et al., 2021; Albright et al., 2024). For example, Shuval (2003) estimated that ~120 million cases of gastroenteritis are reported each year globally, primarily attributable to pollution from wastewater. Shuval (2003) and Wiegner et al. (2021) reported that sewage contamination of surface and groundwater systems significantly harms freshwater ecosystems, including rivers and wetlands. These impacts result in an estimated global economic loss exceeding $16 billion annually due to waterborne diseases contracted during recreational activities, along with an additional $32 billion in losses to ecosystem services globally. Humphrey et al. (2011) posited that, among other pollutants in surface and groundwater harming human health, pathogens such as *Escherichia coli (E. coli)* are particularly concerning. With typical *E. coli* contamination found in watersheds being from birds and mammals (humans, livestock, wildlife, etc.) (Schwab et al., 2014; Borel et al., 2015; Humphrey et al., 2024), *E. coli* is considered a fecal indicator bacterium, meaning its presence indicates potential contamination from other harmful fecal bacteria that are not as easily monitored. The United States Environmental Protection Agency (EPA, 2023) stated the highest level of contamination for *E. coli* presence in watersheds is zero colony-forming units per 100 ml, implying even a trace of *E. coli* in watersheds is considered hazardous. *E. coli* and other fecal bacteria become a significant pollutant in watersheds if not treated effectively in the drain fields of septic systems (White et al., 2021; Osińska et al., 2023).

Watersheds are a key component of environmental and economic quality as they remain important for recreation, drinking water, agriculture, and forestry. However, to safely use these watersheds for their intended purposes, a regular assessment of HBC and water quality is imperative. In Texas, the Texas Commission on Environmental Quality (TCEQ) conducts a comprehensive assessment every 2 years to evaluate water quality, ensuring water bodies meet their designated uses with minimal adverse effects on the ecosystem (TCEQ, 2012). Schwab et al. (2014) noted the assessment encompasses multiple key parameters: “(1) dissolved oxygen standards crucial for supporting aquatic life, (2) *E. coli* standards essential for ensuring safe recreational use, and (3) nitrate and chlorophyll-a screening levels to monitor and maintain overall water quality for general uses” (p. 7). Of the 568 total impairments observed in Texas-based watersheds, 45% are caused by elevated *E.coli* levels (TCEQ, 2012). According to the Texas Integrated Report for Clean Water Act Sections 305(b) and 303(d) in 2010, *E. coli* levels in the East Texas Attoyac Bayou watershed exceeded the standard for its designated purpose and were declared unhealthy for public use in 2004 (TCEQ, 2012; Schwab et al., 2014).

Watersheds are critical for providing drinking water, recreational opportunities, and sustainable livelihoods (EPA, 2023). “We all live in a watershed, the area that drains to a common waterway, such as a stream, lake, estuary, wetland, surface, and groundwater, aquifer, or even the ocean, and our actions can directly affect it” (p. 1). This suggests the economic reliance on clean water and healthy watersheds is substantial, with over $450 billion in grain and fiber production, accounting for ~0.43% of the global GDP, equating to $56.25 per person. Furthermore, Tamang et al. (2022) noted that an estimated 20% of septic system owners in Australia and the United States, 26% in Europe, and 14% in Canada depend on onsite domestic wastewater treatment and disposal systems for treating water effluent (Huffman et al., 2018; White et al., 2021; Brewton et al., 2022). Thus, protecting watersheds from pathogen effluents, such as onsite wastewater treatment systems, is essential.

Adegoke and Stenstrom (2019) noted that failing septic systems can impact groundwater due to poor-quality design, geology, density of the septic tanks, and inadequate maintenance habits. In 2024, Humphrey et al. indicated reactions (e.g., physical, chemical, and biological) occur during the process of wastewater effluent treatment within the drain field soil, and contamination of surface and groundwater can occur if septic systems are not properly designed and maintained. Thus, they imply septic system owners should manage and maintain their septic systems to mitigate the harmful impact of septic effluents. Many innovations have been developed to prevent septic system failure through new designs (e.g., aerobic systems) and siting requirements before permitting. However, the affordability of these systems, especially in rural areas, is of great concern (Sharma et al., 2022) because of the insufficient resources for replacement of older systems and maintenance (White et al., 2021).

Currently, the EPA provides septic system owners with guidelines for care and maintenance. The average household system undergoes professional maintenance every 3 years (EPA, 2024). They also recommend that systems with electronic float switches, pumps, or mechanical components (aerobic systems) should be inspected annually. The major factors influencing the recommended frequency of pumping are household size, total wastewater generated, volume of solids, and septic tank size. Some permitting jurisdictions may also require a service contract to ensure septic system maintenance is performed on more complex systems (e.g., aerobic systems) (EPA, 2024).

Given that watershed contamination is primarily linked to human activities (Greaves et al., 2013), it is reasonable to assert that a fundamental change in human behavior is necessary to reduce contamination and promote a healthy environment. To examine factors that influence septic system owners’ decisions to improve septic system maintenance and protect watershed health through reduced *E. coli levels*, we used Ajzen’s (1985, 1991) theory of planned behavior (TPB).

**Fig. 1:**
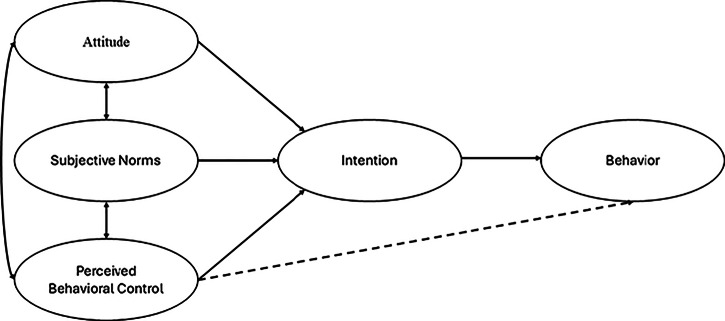
Ajzen’s (1985, 1991) Theory of Planned Behavior

Ajzen’s (1985) TPB is a psychological model comprising three key components: attitude, subjective norms, and perceived behavioral control (Ajzen, 1985, 1991). The three components work together and shape behavioral intention. Attitude refers to an individual’s positive or negative evaluation of a certain behavior (Ajzen, 1985, 1991). The perceived social pressure to perform a behavior (or not) is known as subjective norms (Ajzen, 1985, 1991). Perceived behavioral control is an individual’s belief about their ability to perform a behavior (Ajzen, 1985, 1991). This provides a model for understanding how beliefs about behavior, social pressure, and perceived behavioral control influence engagement in a particular behavior (see Fig. 1).

TPB has been exhaustively applied in many disciplines to investigate human behavior, including healthcare (e.g., Hardeman et al. 2002, Alhamad and Donyai 2021, Shmueli 2021); household settings; and environmental conservation (Greaves et al., 2013; Sarge et al., 2018). For example, a systematic review of pro-environmental behavior involving 699 articles revealed a significant research focus on ecologically friendly behavior at home, such as recycling, composting, and energy conservation, which underscores the importance of domestic actions in promoting environmental sustainability (Concari et al., 2020).

**Fig. 2:**
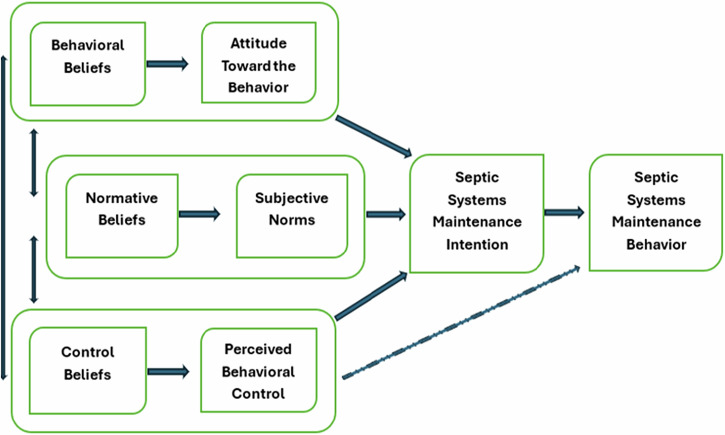
The Theory of Planned Behavior in the context of septic systems. Note. Adapted from Ajzen (1985, 1991)

Ajzen (1985, 1991) first introduced TPB after adding the construct of perceived behavioral control to the earlier theory of reasoned action. Attitude toward the behavior is the first component of the TPB, which, in our study’s context, explains how septic system owners view the behavior of septic system maintenance. This construct underpins the expectations regarding the behavior resulting in a particular desired outcome (Ajzen, 2020). In our study, subjective norms were defined as a means to predict and explain behavior regarding septic system maintenance, influenced by peer pressure. People may engage in this behavior due to the societal values or personal connections they have with their peers, leading them to meet their peers’ expectations. Perceived behavioral control, the third construct in our study, relates to septic owners’ belief in their ability to carry out a behavior, considering their confidence and control over that behavior. Although perceived behavioral control can influence direct change in actual behavior (septic system maintenance behavior), it can also influence change indirectly from the behavioral intention (septic system maintenance intention). Consequently, septic system owners’ intention to conduct septic system maintenance routinely depends on their attitude toward septic systems, subjective norms for conducting septic system maintenance, and perceived behavioral control of septic system maintenance (see Fig. 2).

The purpose of our study was to use TPB to predict septic system owners’ intentions to conduct routine septic system maintenance and identify which constructs in the TPB model influence septic system maintenance behaviors. To achieve this, we had three research questions:

1. What are the characteristics of septic systems within the Attoyac Bayou watershed area?
2. How did septic system owners in the Attoyac Bayou watershed maintain their systems?
3. How do attitudes, subjective norms, and perceived behavioral control predict septic systems maintenance behaviors?

## Methods

Our study is part of a larger study, so similar methods may exist elsewhere.

### Study Site

**Fig. 3:**
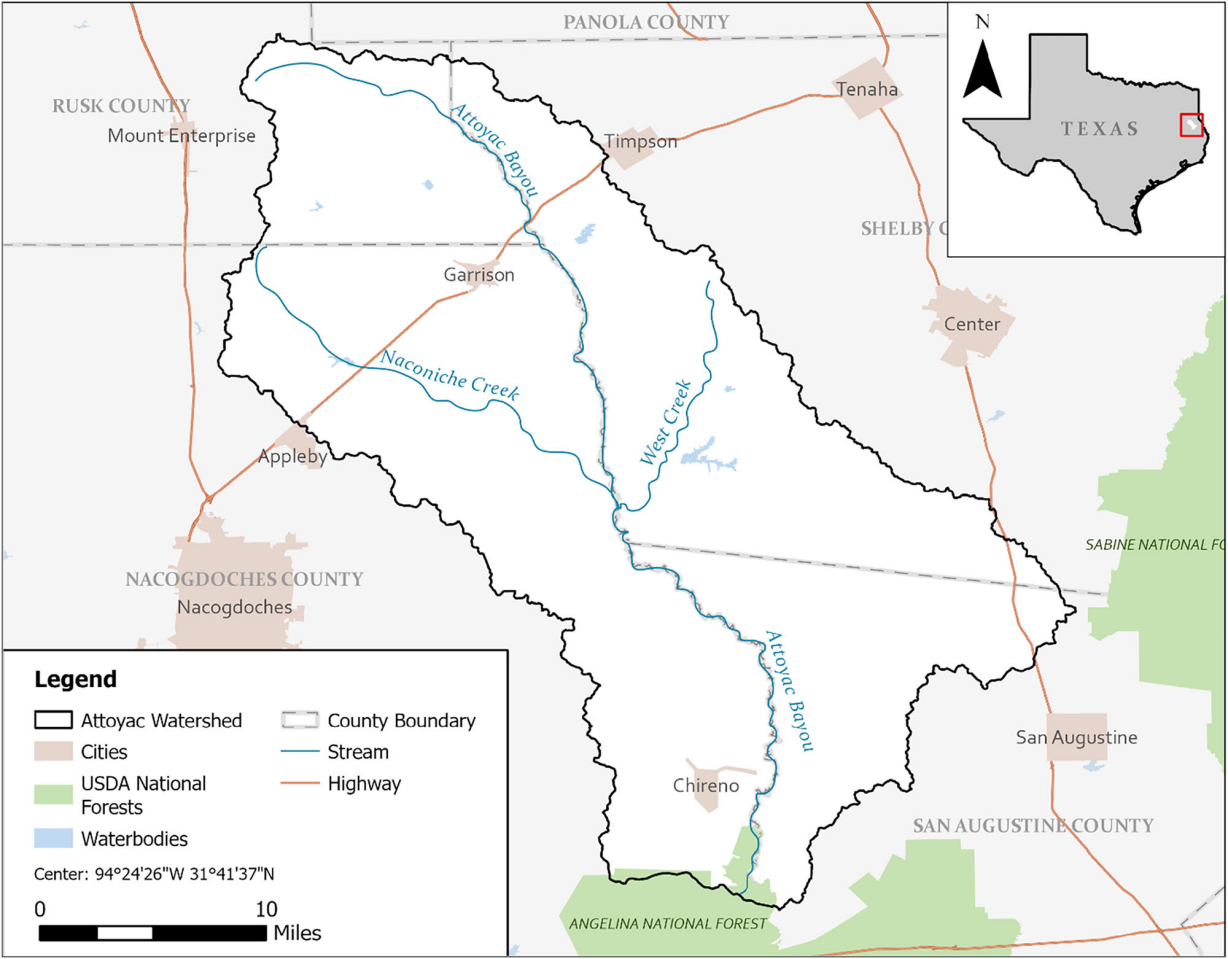
Attoyac Bayou watershed map

We explored the management practices of septic system owners in the Attoyac Bayou watershed in East Texas (Fig. 3). The river basin, covering 354,629 acres, begins in Rusk County next to US Highway 84 and travels through Nacogdoches County, San Augustine, and Shelby Counties before discharging into the Sam Rayburn Reservoir south of Farm-to-Market Road 103. The Attoyac Bayou sustains general usage, contact recreation, and aquatic inhabitants, and serves as a public water supply. Due to water quality degradation and elevated *E. coli* levels above acceptable standards, a watershed protection plan was developed for the Attoyac Bayou watershed to improve water quality. In the water protection plan, failing septic systems were identified as a major contributor to elevated *E. coli* levels; therefore, we sought to explore septic system maintenance in the watershed to identify potential opportunities for improvement.

### Study Design

To conduct our study, we designed a questionnaire to collect information about septic system characteristics, homeowner maintenance histories, sociodemographic characteristics, and intentions, norms, controls, and attitudes related to septic systems based on the TPB model.

### Sample

We derived our sample through purposive sampling to obtain a list of parcels with established land use data in the Attoyac Bayou watershed. Parcels inside of city boundaries and sewer service areas were excluded (Texas Department of Transportation, 2022; Public Utility Commission of Texas, 2022). The remaining parcels outside of sewer service areas were used to form the participant mail list, with the assumption that they potentially contained structures with septic systems attached. Records of septic system permits were unavailable and current geospatial methods to identify septic system locations are extremely limited, so this approach was the most feasible option.

For the demographic characteristics, we conducted descriptive analyzes (i.e., frequencies and percentages). The frequency refers to the number of observations or occurrences where respondents selected a specific value or category, and the percentage is the proportion of a particular frequency relative to the total number of data points, expressed out of 100. We collected responses from 454 individuals, giving us an overall response rate of 13.42%. However, 204 responses did not meet filter requirements or were returned blank. Thus, the final count was 252 responses with a response rate of 7.45%. Most respondents were not first-time septic system owners (*f* = 162; 64.29%) and had lived in their homes for more than 20 years (*f* = 137; 54.37%). Participants’ ages varied with many in their 70 s (*f* = 79; 32.51%), 60 s (*f* = 74; 30.86%), or 80 s (*f* = 47; 19.34%).

### Instrumentation

The Texas A&M University Institutional Review Board approved all study materials (IRB2021-1576M). A survey package was sent to participants consisting of a cover letter, an informed-consent information sheet, the questionnaire, and a pre-paid postage business reply envelope. There was a filter question placed at the beginning of the survey to confirm: (a) the survey respondent was the homeowner, (b) the home has a septic system, and (c) the home’s septic system was in the project area. If a respondent did not meet the question’s requirements, they were asked to end the questionnaire.

For questions related to the perceptions of septic system maintenance, statements were divided into normative perceptions, controls, attitudes, and intentions, following the TPB model (Ajzen, 1991). A five-point scale from *strongly disagree* to *strongly agree* was used for responses. Table 1 outlines the statements representing the constructs.

**Table 1:** Statements in the TPB perceptions of maintenance model

Constructs	Statement
Attitudes	
*Attitude 1*	I think maintaining my septic systems is helpful for the environment.
*Attitude 2*	I think maintaining my septic system protects water quality in water sources nearby.
*Attitude 3*	I think maintaining my septic system benefits public health.
*Attitude 4*	I think having a well-maintained septic system benefits my health.
Norms	
*Norm 1*	It is important to my neighbors that I maintain my septic system.
*Norm 2*	Septic system owners should know how to maintain their septic systems.
*Norm 3*	The government is responsible for ensuring septic systems are maintained.
*Norm 4*	I have a responsibility to my community to maintain my septic system.
Control	
*Control 1*	I know what needs to be done for septic system maintenance.
*Control 2*	I have the time to maintain my septic system.
*Control 3*	I have the financial resources to maintain my septic system.
*Control 4*	I have the skills to identify a failing septic system.
*Control 5*	I feel comfortable making repairs to my septic system myself.
Intentions	
*Intention 1*	I will have my septic system inspected within the next five years.
*Intention 2*	I will have my septic system pumped within the next five years.

### Data Collection

To collect data, we obtained addresses from the Texas Geographic Information Office (TxGIO) database and verified them using third-party services against the National Change of Address database, which contains public appraisal district tax parcel and land use data, to guarantee accuracy and up-to-date information. Duplicate addresses and owner names were excluded to ensure accuracy, and any confidential information was properly stored and only accessed by those included in the IRB. We used a modified tailored design method (Dillman et al., 2014) to distribute the survey package, including pre-notice and thank-you postcards 3 days before and after the survey package to encourage participation and increase the response rate. Survey responses were collected for 30 days after the initial distribution of the pre-notice card. The study received 252 responses from 3383 residents (7.45%), which is considered acceptable for a topic-specific community survey (Brehm et al., 2013).

### Data Analysis

To analyze Research Question 1 (RQ1) and Research Question 2 (RQ2), we conducted a descriptive analysis (i.e., *f*, *%*). For RQ1, we sought to determine the characteristics of respondents’ septic systems (i.e., type, age). As for RQ2, we sought to describe how septic system owners in the Attoyac Bayou watershed maintain their system, looking closely at the service contract, last pump-out, pump-out frequency, last inspection, and inspection frequency. Table 2 presents questions used to measure the septic system characteristics.

**Table 2:** Characteristics of septic systems and maintenance history questions

Variable	Questions
Septic system type	What type of septic system does your home have?
Septic system age	How old is the current septic system?
Service contract	Do you have a service contract with a professional maintenance provider for the septic system?
Last pump out	When was the septic system last pumped out?
Pump out frequency	How often do you have the septic system pumped out?
Last inspection	When was the septic system last inspected by a professional?
Inspection frequency	How often do you have a professional inspect the septic system?

For Research Question 3 (RQ3), we investigated how attitudes, subjective norms, and perceived behavioral control predict septic systems maintenance behavior. An exploratory and confirmatory factor analysis (CFA) was conducted to identify the concepts underlying septic system owners’ behavior towards maintenance and to evaluate the TPB model for reliability, validity, and relationships among latent constructs of the questionnaire developed from these concepts (Forza, 2002).

Data were analyzed using JASP version 0.18.3 (JASP Team, 2024). The data were randomly split into two equal subsamples of 126 responses each: one for developing the questionnaire model using exploratory factor analysis (EFA) and the other for testing it with CFA. Cronbach’s alpha and split-half reliability adjusted with the Spearman–Brown coefficient were used to measure internal consistency and reliability of TBP item construct statements.

Subsample 1 was used for reliability testing and EFA. The reliability of scales was analyzed with Cronbach’s alpha and the Spearman–Brown coefficient (Eisinga et al., 2013). Cronbach’s alpha values closer to one indicate strong reliability though values extremely close to one can indicate redundancy in measurement (Taber, 2018). The Spearman–Brown coefficient is more appropriate for scales containing two items, such as the Intentions scale (Eisinga et al., 2013). For our study, Cronbach’s alpha values were considered acceptable at a value greater than or equal to 0.70, and Spearman–Brown coefficients were acceptable at greater than or equal to 0.50 for moderate correlation (Ratner, 2009; Hair et al., 2011). In the initial EFA, data were evaluated with Bartlett’s test of sphericity and the Kaiser–Meyer–Olkin (KMO) test’s Measure of Sampling Adequacy (MSA) to determine suitability for factor analysis (Bartlett, 1951; Kaiser, 1970; Cureton and D’Augustino, 1983). The EFA was conducted using a principal axis factoring approach with varimax rotation. Kaiser’s criterion (Kaiser, 1960) of Eigenvalues greater than one was applied to determine the number of factor components accepted for modeling.

Subsample 2 was used for confirmatory and structural analysis of the model developed with EFA results of Subsample 1. The fit indices used in the CFA was the Comparative Fit Index (CFI) and standardized root mean square residual recommended by Hu and Bentler (1999) for tests of models with approximate sample sizes of 250 responses or less. Additionally, the AVE was evaluated using a CFA to determine construct validity. The composite reliability of each construct in the CFA was calculated using Coefficient omega (Hair et al., 2011).

### Limitations

There are limitations to our study. First, we cannot generalize our results due to the inadequacy of item questions (indicators), sample size, and non-random sampling methods used in the development of the participant mailing list. This means we cannot generalize our results to American septic system owners outside of the Attoyac Bayou in East Texas who participated in this study. Second, in our analysis, some TPB constructs showed alpha loading coefficients lower than ideal due to the small number of items measuring each construct. Although the items were consistent and reliable, a larger sample size would have increased the variance of all TPB constructs. We had a 13.42% response rate, but not all the data were usable, which may have impaired the validity of the findings. Despite this, there were no demographic differences between the response and non-response groups, implying the sample was adequate for analysis. Although we did not have demographic data for non-respondents, the sampling frame was drawn from a homogeneous population, suggesting minimal demographic variation.

## Results

### RQ1: What are the Characteristics of Septic Systems Within the Attoyac Bayou Watershed Area?

Similar to Table 3, we conducted descriptive statistics (i.e., frequencies and percentages) for Table 4. More than half (*f* = 131, 52%) of septic system owners use conventional septic systems with 45% (*f* = 113) of the septic systems in the watershed being at least 20 years old (Table 4).

**Table 3:** Demographic characteristics (*N* = 252)

Characteristic	Frequency *(f)*	Proportion *(%)*
First time septic owner (*n* = 252)		
No	162	64.29
Yes	90	35.71
Home tenure (*n* = 252)		
Less than 3 years	16	6.35
3–5 years	14	5.56
6–10 years	35	13.89
11–20 years	50	19.84
20+ years	137	54.37
Age (*n* = 243)		
20–29	2	0.82
30–39	4	1.65
40–49	12	4.94
50–59	20	8.23
60–69	74	30.86
70–79	79	32.51
80–89	47	19.34
90–99	4	1.65
100+	1	0.41

Sample size varied across variables due to non-response

**Table 4:** Descriptives of septic systems characteristics within the Attoyac Bayou watershed (*N* = 252)

Constructs	Frequency *(f)*	Proportion (%)
Septic system type		
Conventional	131	52
Aerobic	80	32
Not sure	24	9
Others	17	7
Septic system age		
>20 years	113	45
11–20 years	60	24
< 5 years	37	15
6–10 years	31	12
Not sure	11	4

### RQ2: How have Septic System Owners in the Attoyac Bayou Watershed Maintained their Systems?

We found 82% (*f* = 206) of respondents do not have a service contract for their septic systems, and 25% (*f* = 64) have never pumped out their septic systems. We also found 26% (*f* = 65) have never had their septic system inspected. Meanwhile, 35% (*f* = 87) of the population only have their septic systems inspected when the system is not functioning properly or damaged (Table 5).

**Table 5:** Descriptives of septic system maintenance within the Attoyac Bayou watershed

Item	Frequency *(f)*	Proportion (%)
Service contract (*n* = 250)		
No	206	82
Yes	44	18
Last pump-out (*n* = 252)		
Never	64	25
1–3 years	61	24
>5 years	42	17
<1 year	34	13
4–5 years	31	12
Not sure	20	8
Pump out frequency (*n* = 252)		
Never	75	29
Problem only	66	26
Every 3–5 years	65	26
Every 6–10 years	24	9
Every 2 years	12	5
Once a year	10	4
Last inspection (*n* = 251)		
Never	65	26
1–3 years	50	20
<1 year	46	18
Not sure	40	16
>5 years	36	14
4–5 years	14	6
Inspection frequency (*n* = 248)		
Problem only	87	35
Never	74	30
Every 3–5 years	39	16
Once a year	27	11
Every 2 years	12	4
Every 6–10 years	9	4

Sample size varied across variables due to non-response

### RQ3: How do Attitudes, Subjective Norms, and Perceived Behavioral Control Predict Septic Systems Maintenance Behaviors?

Upon analysis of the *Norms* construct using Cronbach’s alpha, the item *Norm 3* was dropped from the analysis, which increased the Cronbach’s alpha value for the *Norms* construct from 0.57 to 0.74. The *Controls*, *Attitudes*, and *Intentions* constructs had acceptable composite Cronbach’s alpha values greater than 0.70.

In the EFA, Bartlett’s test for Subsample 1 was statistically significant (*χ*^2^ = 870.131, *df* = 78, *p* < 0.001), indicating differences between correlations of variables in the data that make it suitable for factor analysis. The KMO test indicated an overall MSA value of 0.80 with individual variable MSAs > 0.50, indicating model variables would yield reliable factors for further analysis (Kaiser, 1974; Hair et al., 2006). The EFA of Subsample 1 revealed four factors representing 75.1% of variance for the 13 variables. Item *Norm 4* was split between constructs *Norms* and *Attitudes*; however, it loaded more heavily on the *Norms* factor. Therefore, we placed it as a *Norms* for subsequent analyzes. Additional analysis revealed that *Control 5* had a poor fit within the *Controls* factor and overall model. Therefore, *Control 5* was removed from the model. The final model Eigenvalues and factor loadings of each construct analyzed in Subsample 1 EFA are presented in Table 6.

**Table 6:** Exploratory factor analysis of subsample 1

Constructs	Eigenvalue	Factor loading
Attitudes	5.076	
*Attitude 1*		0.836
*Attitude 2*		0.853
*Attitude 3*		0.703
*Attitude 4*		0.823
Norms	1.520	
*Norm 1*		0.503
*Norm 2*		0.625
*Norm 4*		0.773
Controls	2.521	
*Control 1*		0.839
*Control 2*		0.702
*Control 3*		0.667
*Control 4*		0.700
Intentions	1.053	
*Intention 1*		0.949
*Intention 2*		0.676

See Table 2 for full statement descriptions

Based on the EFA of subsample 1 and previous literature, we know the *Intentions* factor should represent a second-order factor with input from the *Norms*, *Controls*, and *Attitudes* factors. The second-order CFA of Subsample 2 (Table 7) using the Maximum Likelihood Estimator yielded a statistically significant chi-square statistic (*χ*^2^ = 117.112, *df* = 59, *p* < 0.001). However, due to the sensitivity of the chi-square statistic to sample size, we reviewed additional indicators to determine an acceptable model fit. The combination of indices: CFI (0.956) and standardized root mean squared residual (SRMR) (0.048) indicated acceptable construct validity for the model (CFI > 0.95 and SRMR < 0.08), according to Hu and Bentler (1999). Reliability for each construct met composite reliability of >0.70 (Hair et al., 2011). Additionally, the average variance extracted (AVE) of each construct was above 0.50, indicating acceptable convergent validity (Hair et al., 2011).

**Table 7:** Confirmatory factor analysis of subsample 2 with standardized estimates

Construct	Composite reliability	Factor loading	*z*-value
Attitudes	0.955		
*Attitude 1*		0.937	–
*Attitude 2*		0.935	20.216
*Attitude 3*		0.861	15.525
*Attitude 4*		0.946	21.177
Norms	0.814		
*Norm 1*		0.776	–
*Norm 2*		0.897	10.606
*Norm 4*		0.760	8.822
Controls	0.881		
*Control 1*		0.869	–
*Control 2*		0.830	11.348
*Control 3*		0.724	9.257
*Control 4*		0.820	11.137
Intentions	0.867		
*Intention 1*		0.981	–
*Intention 2*		0.776	6.186

We confirmed the discriminant validity using the Fornell-Larcker criterion, requiring the square root of the AVE (SQAVE) to be greater than the correlation between latent constructs (Table 8).

**Table 8:** Matrix of SQAVE and construct coefficient variances

Construct	AVE	Attitudes	Norms	Controls
Attitudes	0.839	**0.916**		0.357
Norms	0.639	0.483	**0.799**	–
Controls	0.648		0.393	**0.805**

Bolded numbers = SQAVE

## Conclusions

The findings from the current study make clear that septic system maintenance is not simply a technical issue but a behavioral and social one with broad environmental and public health consequences. Our factor analyzes showed attitude is the strongest driver of maintenance intentions, while perceived behavioral control and subjective norms play smaller roles. Septic system owners who hold and express a positive attitude toward maintenance are significantly more likely to inspect and pump their systems regularly. These proactive behaviors help reduce *E. coli* contamination and contribute to the protection of freshwater ecosystems. At the same time, intention often fails to become action when cost, limited knowledge, weak enforcement, and logistical barriers intervene.

## Discussion

The U.S. EPA reported that, in low-density (rural) communities, conventional septic systems are often preferred due to their relatively low cost when compared to other types of wastewater management systems (Massoud et al., 2009). Recent research indicates that around 16% of U.S. households are connected to standard septic tanks, underscoring the significant reliance on conventional systems for wastewater management in many regions (Yu et al., 2024). Septic systems represent a significant capital investment and require substantial ongoing maintenance and operational costs. Consequently, many rural and peri-urban areas opt for conventional septic systems to mitigate costs associated with inspecting and maintaining centralized wastewater systems.

However, the age of the system, lack of maintenance, and improper management have been linked to septic system failures (Bremer and Harter, 2012; Ravi and Johnson, 2021), and overall wastewater treatment effectiveness (Adegoke and Stenstrom, 2019; Humphrey et al., 2024). According to the U.S. EPA (2019), septic system owners should have their systems inspected every 3 years and ensure malfunctioning units are repaired promptly. Maintenance frequency is influenced by household size, as larger households generate more wastewater and solids. For example, a household of five typically requires septic tank pumping every 3 to 5 years, consistent with EPA recommendations. Regular pumping every 3 to 5 years also helps maintain proper system performance and reduces *E. coli* levels in wastewater discharged into nearby watersheds (Fizer et al., 2018). Ravi and Johnson (2021) stressed that regular field inspections are critical to preventing failures. Establishing a service contract with a trained and knowledgeable septic system professional not only helps prevent system failures from physical constraints but also extends the system’s lifespan and enhances treatment efficiency (Sharma et al., 2022). By keeping the system in good working condition through regular inspections and maintenance, homeowners can avoid costly repairs, protect their property, and contribute to a cleaner and healthier environment.

The EFA showed that TPB constructs accounted for 75.1% of the variance in septic system owners’ maintenance behavior (Ajzen, 1985, 1991). Among these constructs, attitude accounted for the highest portion of variance, suggesting it has the strongest influence on maintenance behavior. This indicates that, when owners view septic system maintenance as beneficial for protecting their property and the environment, they are more likely to engage in recommended practices such as regular inspection and pumping. This is supported by Hristova et al. (2025) who found that environmental attitudes, including verbal, affective, and actual commitment, were the strongest predictors of general ecological behavior. Other researchers have similarly shown that positive attitudes predict greater engagement in environmental and health-protective behaviors (Kaiser et al., 2005; Ajzen and Fishbein, 2018). In contrast, perceived behavioral control and subjective norms were less dominant, suggesting these factors play a smaller role in shaping intentions to perform maintenance tasks. This highlights the importance of strengthening and promoting positive attitudes toward septic system care to encourage consistent and responsible maintenance behavior among homeowners, ultimately supporting long-term environmental and public health protection.

In the CFA, all constructs (attitude, subjective norms, perceived behavioral control) indicated strong internal consistency, demonstrating the items reliably and validly measure their respective constructs within the context of septic system maintenance (Ajzen, 2020). Loadings for attitude showed a strong relationship between the variable and the factors, implying that owners generally have a positive outlook toward maintenance. They recognize that routine maintenance improves public health, water quality, and environmental sustainability, consistent with prior literature (Khatri and Tyagi, 2014; Rahman et al., 2021). These findings reaffirm the central role of attitude in shaping maintenance behavior and underscore the need for interventions that foster positive perceptions and awareness about proper septic system care.

Within the TPB model, subjective norms reflect the influence of peers and societal expectations on behavior. In our study, owners were moderately influenced by a sense of community obligation to maintain their systems routinely (Greaves et al., 2013). Perceived behavioral control showed a moderate to strong relationship with maintenance intentions, indicating that owners possess the behavioral control and knowledge to perform necessary tasks. A favorable perception of behavioral control significantly influences intention to maintain septic systems (Ajzen, 1985). Our study showed owners demonstrated a strong intention to inspect their systems, reflecting their recognition of the importance of maintenance and their confidence in managing the required tasks. When owners believe they have the necessary skills, time, and resources, their motivation to act is strengthened, reinforcing the connection between intention and actual behavior.

Ajzen’s (1985, 1991) TPB posits that individuals with strong behavioral, normative, and control beliefs about a behavior are more likely to develop intentions to perform that behavior and believe they can carry it out. In our study, septic system owners expressed intentions to maintain their systems but were only moderately influenced by societal values or personal control to take concrete actions. Their subjective norms and controllability regarding routine maintenance were insufficient to translate reliably into maintenance behavior. These gaps may be driven by several factors, including high cost of maintenance and upgrades, limited knowledge of proper practices, low awareness of importance, lengthy maintenance processes, and weak policy enforcement (White et al., 2021; Albright et al., 2024).

Given that adequate maintenance is further constrained by resource limitations, management challenges, and regulatory factors (White et al., 2021), it is essential to foster positive attitudes and increase awareness among system owners. Interactive educational interventions, such as hands-on workshops, community demonstration projects, and digital awareness campaigns, can improve understanding of the health, property, and environmental benefits of septic system care. These interventions also extend beyond ownership and maintenance, supporting broader watershed protection by reducing nutrients and bacterial contamination in local waterways (Albright et al., 2024). For instance, targeted education about buffer zones, stormwater management, and runoff mitigation can help safeguard watersheds.

### Recommendations

Overall, these results suggest a multifaceted approach. First, we recommend that interventions must prioritize shaping positive attitudes through practical, locally tailored education and communication that demonstrates direct benefits to health, property value, and nearby watersheds. Hands-on workshops, community demonstration projects, and clear digital materials will build understanding and normalize maintenance as a routine household responsibility. Second, policy and financial tools are needed to remove barriers. Targeted subsidies, low-interest loans, or cost-sharing for inspections and upgrades will make maintenance more affordable, while clearer regulations and improved enforcement will help close the gap between intent and behavior. Third, professional services models such as routine service contracts should be encouraged so that technical capacity is accessible and maintenance becomes predictable rather than episodic. Fourth, future research should test integrated interventions using rigorous experimental or longitudinal designs, evaluate cost effectiveness, and explore equity, professional services, and innovative technology. A coordinated strategy offers a practical pathway from individual attitudes to measurable environmental and social benefits. Equally, it calls for investigating factors, such as past behaviors, barriers, and motivations, that impact actual behavioral performance. As such, we can turn positive intentions into consistent action, reduce bacterial and nutrient pollution in watersheds, protect public health, and extend the useful life of septic infrastructure.

## Data Availability

Data used in this study is available upon reasonable request and approval of authors. Shared data may be subject to a data use agreement.
